# Prochlorperazine Withdraws the Delayed Onset Tonic Activity of Unloaded Rat Soleus Muscle: A Pilot Study

**DOI:** 10.3390/life11111161

**Published:** 2021-10-30

**Authors:** Vitaliy E. Kalashnikov, Sergey A. Tyganov, Olga V. Turtikova, Ekaterina P. Kalashnikova, Margarita V. Glazova, Timur M. Mirzoev, Boris S. Shenkman

**Affiliations:** 1 Institute of Biomedical Problems, Russian Academy of Sciences, 123007 Moscow, Russia; vitaliy.kalasxnikov@yandex.ru (V.E.K.); sentackle@yandex.ru (S.A.T.); olga_tur@list.ru (O.V.T.); mochalova_ekaterina@lenta.ru (E.P.K.); bshenkman@mail.ru (B.S.S.); 2 Sechenov Institute of Evolutionary Physiology and Biochemistry, Russian Academy of Sciences, 194223 St. Petersburg, Russia; mglazova@iephb.ru

**Keywords:** soleus muscle, EMG activity, hindlimb suspension, KCC2, NKCC1, prochlorperazine

## Abstract

A gradual increase in rat soleus muscle electromyographic (EMG) activity is known to occur after 3–4 days of hindlimb suspension/unloading (HS). The physiological significance and mechanisms of such activity of motoneurons under unloading conditions are currently unclear. Since hyperactivity of motoneurons and muscle spasticity after spinal cord injury are associated with KCC2 downregulation, we hypothesized that a decrease in potassium (K^+^)/chloride (Cl^−^) co-transporter 2 (KCC2) in motoneurons would be responsible for an increase in soleus muscle EMG activity during HS. We aimed to investigate the effect of prochlorperazine (KCC2 activator) on the electrical activity of rat soleus muscle under HS. Wistar rats were divided into the following groups: (1) vivarium control (C), (2) 7-day HS group (7HS) and (3) 7-day HS group plus intraperitoneal injections of prochlorperazine (10 mg/kg, daily) (7HS + P). Expression of proteins in the motoneurons of the lumbar spinal cord was determined by Western blotting. An electromyogram of the rat soleus muscle was recorded using intramuscular electrodes. KCC2 content after 7-day HS significantly decreased by 34% relative to the control group. HS-induced decrease in KCC2 protein content was prevented by prochlorperazine administration. HS also induced a significant 80% decrease in KCC2 Ser940 phosphorylation; however prochlorperazine did not affect KCC2 phosphorylation. The treatment of the rats with prochlorperazine prevented a HS-induced increase in Na(+)/K(+)/(Cl−) co-transporter 1 (KCC2 antagonist) protein content. In parallel with the restoration of KCC2 content, prochlorperazine administration during HS partially prevented an increase in the soleus muscle tonic EMG activity. Thus, prochlorperazine administration during 7-day HS prevents a decrease in KCC2 protein expression in motoneurons and significantly reduces the level of HS-induced soleus muscle electrical activity.

## 1. Introduction

A number of authors have repeatedly shown that following 3–4 days of mechanical unloading (hindlimb suspension, HS), rat soleus muscle resumes its electromyographic (EMG) activity [1,2,3]. This EMG activity is apparently not associated with reflex responses to some stimuli. The nature of this kind of EMG activity is still unclear. Integrated soleus muscle EMG activity gradually increases during the course of unloading and, by the 14th day of HS, reaches values comparable to those of control animals. This phenomenon was observed in rodent hindlimb unloading experiments simulating the effect of microgravity in which soleus muscle EMG activity was recorded with the help of implanted electrodes. The physiological significance and mechanisms of delayed onset activity of motoneurons under unloading conditions are currently unclear. Obviously, this phenomenon can hardly be regarded as compensatory. Indeed, the emergence of delayed onset electrical (and mechanical) activity of the soleus muscle and further growth of EMG activity during HS is accompanied by a steady progression of the atrophic process in the soleus muscle [3]. However, the elimination of the propagation of action potential via denervation of the soleus muscle during 14-day HS does not result in greater muscle atrophy [4]. In this regard, the phenomenon of increased excitability of motoneurons due to changes in potassium (K^+^)/chloride (Cl^−^) co-transporter 2 (KCC2) is of great interest [5,6]. Decreased KCC2 expression due to spinal cord injury results in a more positive equilibrium potential for Cl^−^, with subsequent change in synaptic input that normally would be inhibitory to one with a more excitatory effect [6]. The inhibitory effects of GABA and glycine become more excitatory due to the positive shift in the equilibrium potential for Cl^−^ [5,6]. This switch can lead to hyperactivity in motoneurons and spasticity [6]. Recently, it was found that increased excitability and activity of motoneurons due to KCC2 downregulation is also observed following peripheral axotomy [7]. It is important to note that equilibrium potential for Cl^−^ is also dependent on the activity of Na(+)/K(+)/Cl(−) co-transporter isoform 1 (NKCC1), a membrane protein that mediates active transport of sodium, potassium, and chloride into cells [8].

Obviously, maintaining the normal level of KCC2—and, hence, the normal chloride ion gradient and motoneuron excitability—depends on a certain level of neuromuscular activity. The elimination of innervation and “switching-off” of the muscle (EMG activity is silent) should help to increase the excitability of the motoneurons. It is natural to assume that this mechanism would underlie the phenomenon of delayed onset electrical activity of the soleus muscle after 3 days of mechanical unloading. To test this assumption, it is necessary to (1) evaluate the level of protein expression of KCC2 during gravitational unloading (rat hindlimb suspension), and if the level of KCC2 expression/phosphorylation decreases, it is necessary to (2) investigate the delayed onset EMG activity of the soleus muscle using a pharmacological agent that can activate the expression of KCC2 during gravitational unloading. Currently, two pharmacological agents are used to upregulate KCC2 in the motoneurons of the lumbar spinal cord, prochlorperazine [9] and CLP257 [10]. These drugs are able to enhance the expression of KCC2 in the motoneurons and decrease hyperreflexia and spasticity after chronic spinal cord injury. Prochlorperazine belongs to the first-generation antipsychotic drugs. Based on data obtained by scanning the drug database (Prestwick Chemical Library^®^) Liabeuf and co-authors (2017) found that prochlorperazine is able to activate KCC2 [9]. It was shown that a single intravenous administration of prochlorperazine not only increases the expression of KCC2 in motoneurons, but also prevents the development of spasticity after spinal cord injury in rats [9]. In the present study, we aimed to investigate the effect of prochlorperazine on the emergence of delayed onset electrical activity of the rat soleus muscle under hindlimb unloading.

## 2. Materials and Methods

### 2.1. Experimental Design

Male Wistar rats (2.5 months of age, 190 ± 10 g, obtained from the Nursery for Laboratory Animals of the Institute of Bioorganic Chemistry of the RAS, Pushchino, Moscow region) were randomly divided into the following 3 groups (*n* = 8 per group): (1) vivarium control (C), (2) 7-day hindlimb suspension (7HS), and (3) 7-day hindlimb suspension plus intraperitoneal injections of prochlorperazine (7HS + P). Mechanical unloading was performed via tail suspension (hindlimb suspension), as previously described by Morey-Holton and Globus (2002) [11]. Prochlorperazine dimaleate salt (#P9178; Sigma-Aldrich, St. Louis, MO, USA) was administered twice a day (each injection contained 5 mg/kg in saline + 0.1% DMSO). The applied dose was calculated [12] based on the equivalent human therapeutic dose (112 mg/kg). Rats from the C and 7HS groups received an equivalent volume of saline + 0.1% DMSO without prochlorperazine. The rats were anesthetized with an intraperitoneal (i.p.) injection of tribromoethanol (240 mg/kg, #T48402; Sigma-Aldrich, St. Louis, MO, USA) and the lumbar spinal cord was isolated and frozen in liquid nitrogen until further analysis. Upon completion of the experiment, the rats were sacrificed by i.p. injection of tribromoethanol overdose (750 mg/kg, #T48402; Sigma-Aldrich, St. Louis, MO, USA) followed by cervical dislocation. In order to assess soleus muscle EMG activity, 8 additional rats (4 hindlimb unloaded and 4 hindlimb unloaded rats treated with prochlorperazine) were used. Thus, the total number of rats used in the study was 32.

### 2.2. Electrode Implantation

**Figure 1:**
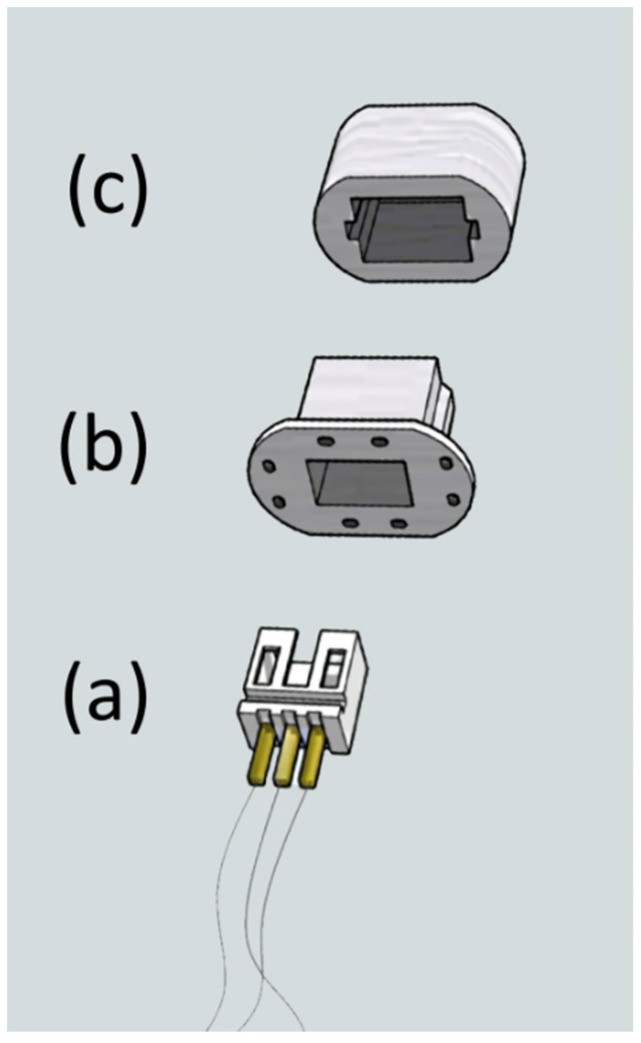
Plug connector with protective cap used in experiment: (**a**) inner part of plug; (**b**) outer part of plug with perforated margins; (**c**) removable protective cap.

An electromyogram of the rat soleus muscle was recorded using intramuscular electrodes. Multi-core stainless steel wires with Teflon PFA coating (A-M Systems, Sequim, WA, USA) were used as electrodes. The wires in the muscle were subcutaneously connected to a plug located on the rat's back. The plugs were made using a 3D printer (Figure 1a). The holes in the wide margins of the plug connector allowed tight attachment of the plug to the skin with a surgical suture (Figure 1b). To protect the plug from possible damage, a removable protective cap was used (Figure 1c).

**Figure 2:**
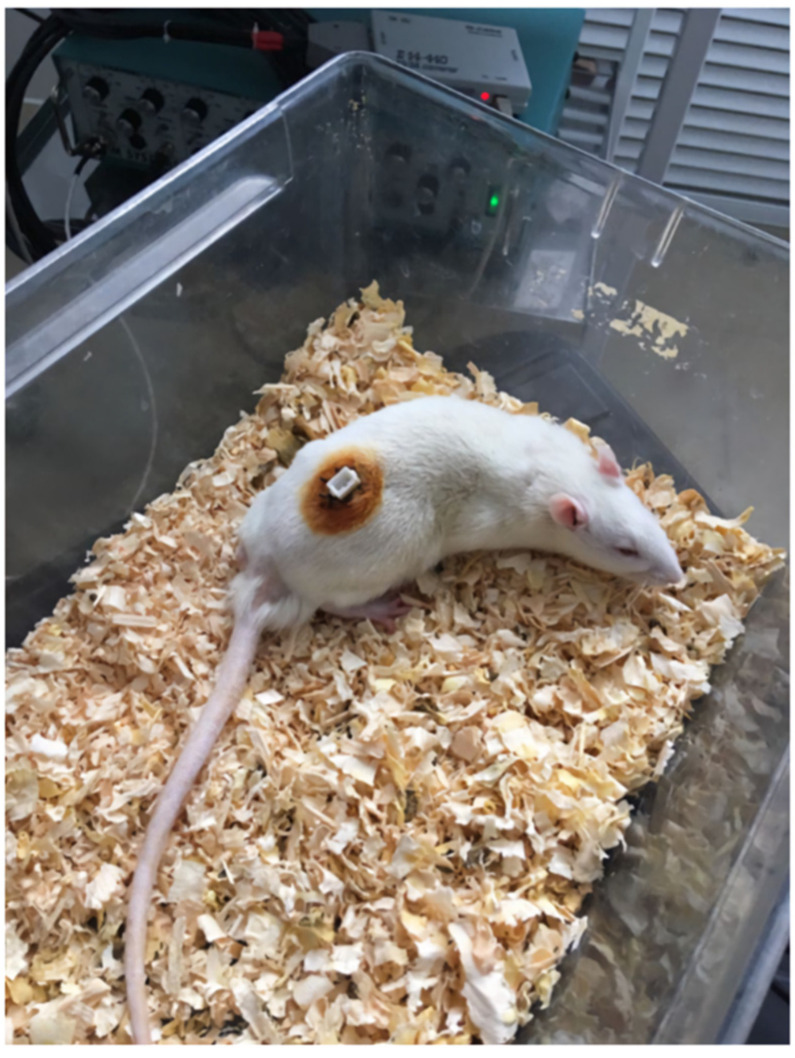
Rat with plug connector attached to lumbar spine.

Electrode implantation was performed under general anesthesia (Zoletil 100, 28 mg/kg + xylazine, 0.28 mL/kg, IM) in compliance with aseptic principles. In order to access soleus muscle, an incision of about 1.5 cm was made on the lateral surface of the hindlimb. An incision of about 1.5 cm above the lumbar spine was made in order to install the plug connector. Bleeding from the vessels was stopped by electrocoagulation. The wires were inserted by threading through a 23-gauge needle being passed through the muscle. A section of the wire with the insulation removed (~1.5 mm) was implanted into the midbelly of the soleus muscle of the right hindlimb. Two wires were inserted parallel to the muscle fibers (~2 mm apart). Each wire was affixed by knots (4-0 Ethilon suture) at its entry and exit from the muscle, while the muscle was able to contract freely. The plug connector with attached silicone oval was inserted into the incision above the lumbar spine and sewn to the skin using 4-0 Ethilon (Figure 2). The incision on the hindlimb was sutured with 5-0 Vicryl, and the skin was sutured with 4-0 Ethilon. After the surgery, each rat received 5 mL of saline solution and an injection of bicillin-3 (120,000 U/kg, subcut) to prevent possible infection. Each animal was under recovery for 7 days before electrophysiological studies were conducted.

### 2.3. EMG Recordings

The electromyographic signal was amplified using an AM-Systems 1700 differential AC amplifier with a sampling frequency of 5 kHz. Initially, the raw signal was filtered using low-cut-off (100 Hz) and high-cut-off (5000 Hz) filters. After that, the received signal was additionally filtered in the PowerGraph program using a bandpass filter 100–1000 Hz. The received signal was processed using the ADC module L-CARD E14-440D and corresponding software (LGRAPH2 and Powergraph 3.3). To evaluate the spectral characteristics of the received signal, fast Fourier transform (FFT) was used, after which additional digital band-pass filters were applied. Analysis of the amplitude of the EMG signal was carried out by root-mean-square (RMS) envelope. Skeletal muscles were not, by any means, stimulated during EMG recordings. Before the initiation of HS, soleus EMG activity in conscious rats was recorded at quadrupedal posture on the floor (rats were placed in small cages and left alone to limit movements) for 15 min 3 times a day during 2 consecutive days. Even though the rats moved occasionally, they were generally sedentary. Analyzed recordings were obtained when the rats were quiet. Then the rats were hindlimb unloaded for 7 days. During unloading conditions, EMG activity was recorded daily (3 times a day for 15 min). Since HS is a well-established in vivo model for hypokinesia, muscular movements were significantly decreased during EMG-recording of the suspended rats. Activity bursts produced by occasional rat movements were not taken into account.

### 2.4. Western Blotting

Frozen lumbar sections of the rat spinal cord were homogenized in ice-cold RIPA buffer (cat #sc-24948; Santa Cruz Biotechnology, Dallas, TX, USA). The samples were then centrifuged for 15 min at 12,000 rpm at +4 °C. The supernatant was separated and the protein concentration was measured by the Bradford method. Sodium dodecyl sulfate–polyacrylamide gel electrophoresis (SDS-PAGE) was performed in 10% polyacrylamide gel using a Bio-Rad mini-system (Bio-Rad, Hercules, CA, USA) for 1 h at a current of 17 mA per gel. The transfer of proteins to the nitrocellulose membrane (Bio-Rad, Hercules, CA, USA) was carried out in a mini Trans-Blot system (Bio-Rad, Hercules, CA, USA) for 2 h at +4 °C and a constant voltage of 100 V. Following the transfer, in order to verify an equal load of protein in all lanes, the nitrocellulose membrane was dyed with Ponceau S. Then the dye was washed off and the membrane was blocked in a 4% solution of powdered milk in PBST (PBS + 0.1% Tween 20) at room temperature for 1 h. Then incubation of the membrane was carried out (15 h, +4 °C) with primary antibodies against KCC2 (1:1000, cat. #07-432; Merck, Burlington, MA, USA), phospho-Ser940 KCC2 (1:1000, cat. #p1551-940; PhosphoSolutions, Aurora, CO, USA), NKCC (1:1000, registry id ab_528406; DSHB, Iowa City, IA, USA), tubulin (cat #ab176560; Abcam, Boston, MA, USA). After that, the membranes were washed in PBST (3 times for 5 min) and incubated with horseradish peroxidase-conjugated secondary antibodies to rabbit immunoglobulins (1:30,000, #111-035-003; Jackson ImmunoResearch, West Grove, PA, USA). Protein bands on the membrane were detected using Clarity Western ECL Substrate (#1705061; Bio-Rad, Hercules, CA, USA). The protein bands were quantified using a C-DiGit Blot Scanner (LI-COR Biotechnology, Lincoln, NE, USA) and Image Studio Digits software. Each gel contained samples from all groups. Protein samples on the same gel were run at least in duplicate. Tubulin content was used as loading control.

### 2.5. Statistical Analysis

Since the normal distribution of the sample was not confirmed in all cases, a nonparametric Kruskal–Wallis test was used to compare the groups with each other. SigmaPlot 12.5 software package was used for statistical analysis. The data are presented as the mean ± standard error of the mean (expressed as a percentage of the mean value of the control group). A *p* value of <0.05 was considered statistically significant.

## 3. Results

### 3.1. KCC1 and NKCC1 Protein Expression in Lumbar Spinal Cord Motoneurons

**Figure 3:**
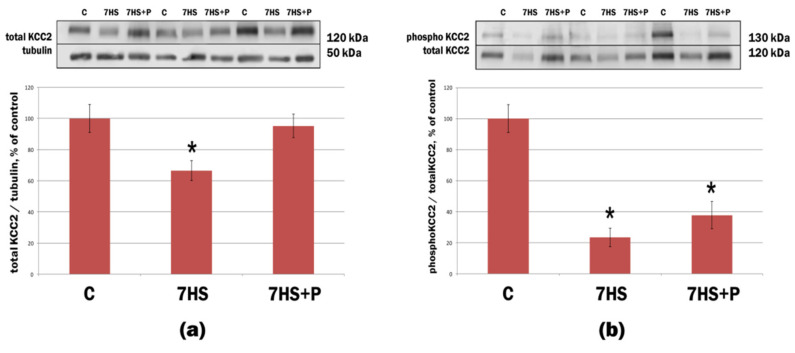
Protein expression of (**a**) total KCC2 and (**b**) phospho-KCC2 (Ser940) in motoneurons of lumbar spinal cord in rats. C: vivarium control; 7HS: 7-day hindlimb suspension; 7HS + P: 7-day hindlimb suspension with daily injections of prochlorperazine, *n* = 8 per group. * *p* < 0.05 vs. C group. Graph shows that 7-day hindlimb suspension/unloading led to a significant decrease in both total content and phosphorylation of KCC2 in spinal motoneurons. This downregulation of KCC2 may be responsible for the emergence of delayed onset electrical activity of rat soleus muscle under hindlimb unloading. Administration of prochlorperazine (a drug known to increase KCC2 expression in motoneurons) during 7-day hindlimb unloading prevented a decrease in KCC2 protein content in spinal motoneurons. We hypothesized that this could prevent the emergence of delayed onset EMG activity in rat soleus muscle.

The content of KCC2 in the motoneurons of lumbar spinal cord in rats following 7-day mechanical unloading significantly decreased by 34% compared to the control group (Figure 3). This HS-induced decrease in KCC2 protein content was prevented by prochlorperazine administration (Figure 3). HS also induced a significant 80% decrease in KCC2 Ser940 phosphorylation relative to the control animals (Figure 3). However, prochlorperazine administration during 7-day HS did not significantly affect KCC2 Ser940 phosphorylation (Figure 3).

**Figure 4:**
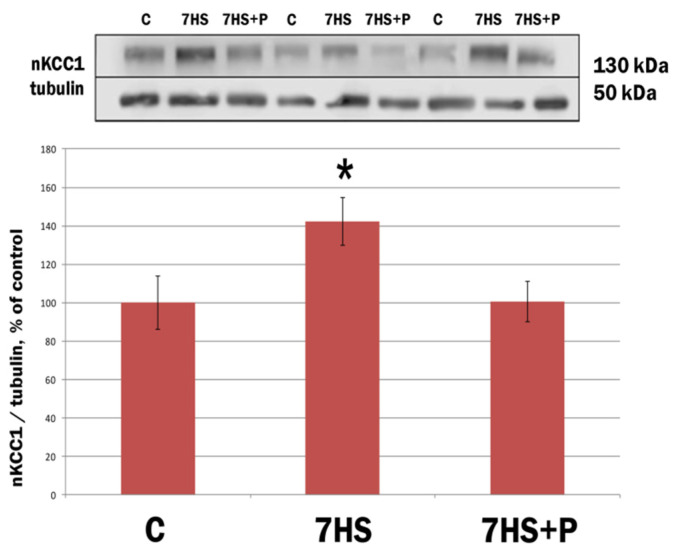
Protein expression of NKCC1 in motoneurons of lumbar spinal cord in rats. C: vivarium control; 7HS: 7-day hindlimb suspension; 7HS + P: 7-day hindlimb suspension with daily injections of prochlorperazine, *n* = 8 per group. * *p* < 0.05 vs. C group. Since NKCC1 is a known antagonist of KCC2, we expected to observe an increase in NKCC1 protein content in spinal motoneurons after 7-day HS; administration of prochlorperazine did prevent such increase.

The protein content of NKCC1 in the motoneurons of lumbar spinal cord in rats after 7-day HS significantly increased by 40% compared to the control group (Figure 4). Treatment of the rats with prochlorperazine during 7-day HS prevented an increase in NKCC1 protein content (Figure 4).

### 3.2. Soleus Muscle EMG Activity

**Figure 5:**
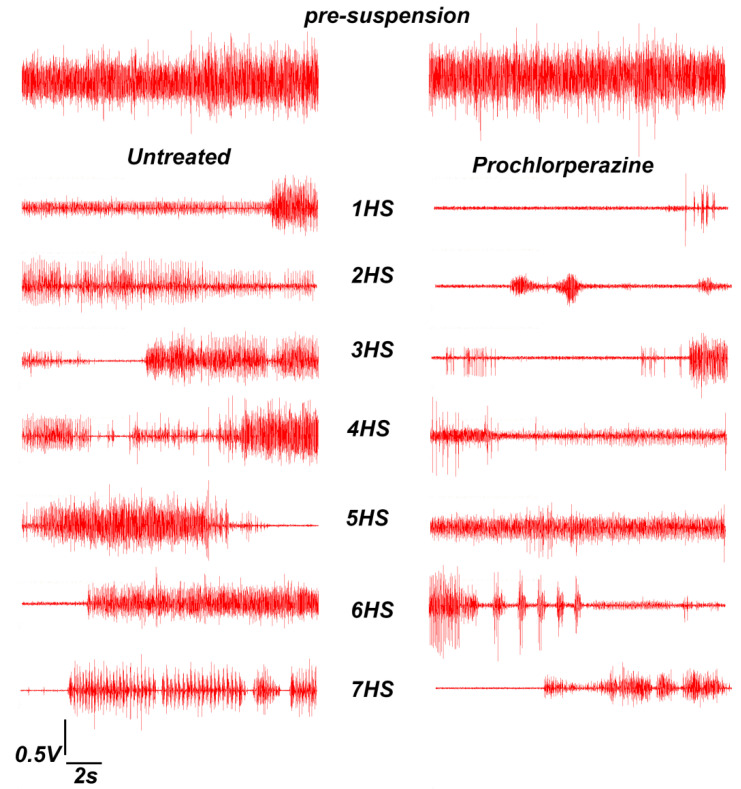
Typical patterns of EMG activity of rat soleus muscle before hindlimb suspension (pre-suspension) and during 7-day HS in the untreated and prochlorperazine-treated rats. 1HS–7HS: days of HS.

**Figure 6:**
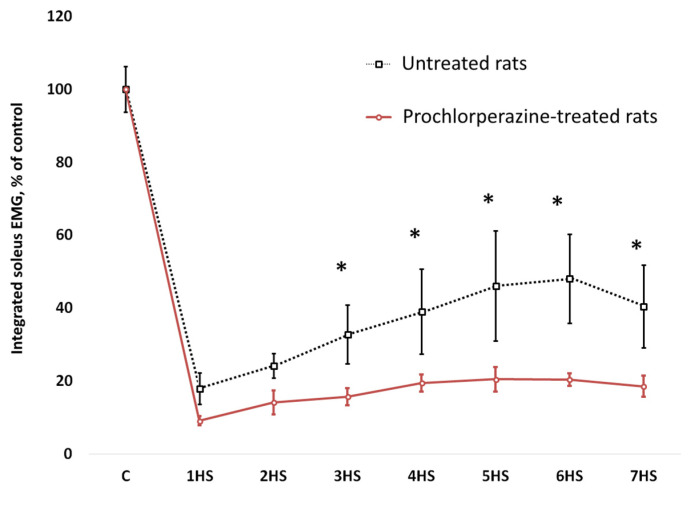
Integrated soleus muscle EMG activity during mechanical unloading (hindlimb suspension). C: pre-suspended rats; 1HS–7HS: days of hindlimb suspension. * *p* < 0.05 vs. rats treated with prochlorperazine during HS. EMG recordings were performed before hindlimb suspension (C) and during 7-day HS in untreated rats and rats treated with prochlorperazine (10 mg/kg daily). As hypothesized, prevention of KCC2 reduction in spinal motoneurons with prochlorperazine prevented the emergence of delayed onset soleus muscle EMG activity during hindlimb unloading.

Representative images of soleus muscle EMG activity obtained before HS (pre-suspension) and during 7-day HS (with and without prochlorperazine treatment) are shown in Figure 5. We observed a sharp decrease in integrated EMG soleus muscle activity by 90% immediately after the start of HS (Figure 6). By the end of the first day of HS, the decrease in integrated EMG soleus activity was 83% compared to the control values (Figure 6). By the 7th day of HS, the decrease in electrical activity of the soleus muscle was only 40% compared to the control group (Figure 6). Prochlorperazine administration during the course of HS partially prevented a delayed onset increase in the inegrated EMG activity of the rat soleus muscle (Figure 6).

**Figure 7:**
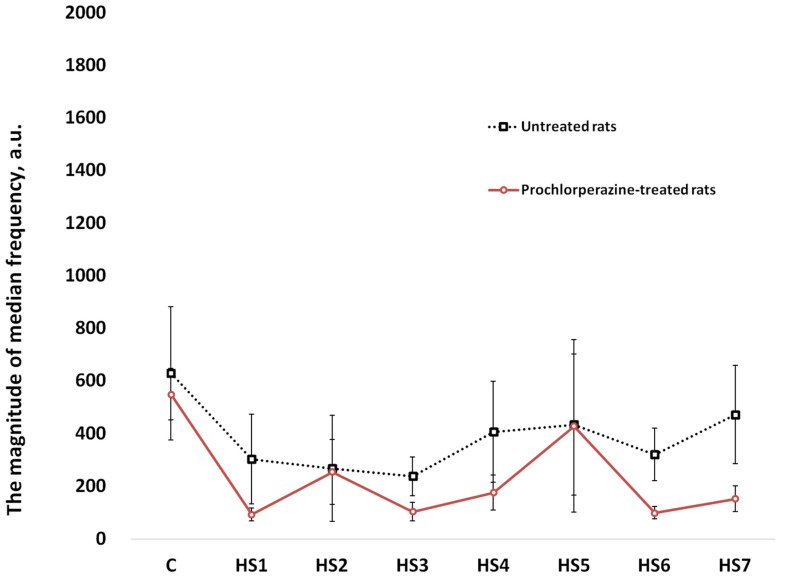
Mean values of the magnitude of the median frequency during mechanical unloading (hindlimb suspension). C: pre-suspended rats; 1HS–7HS: days of hindlimb suspension. EMG recordings were performed before hindlimb suspension (C) and during 7-day HS in untreated rats and rats treated with prochlorperazine (10 mg/kg daily).

**Figure 8:**
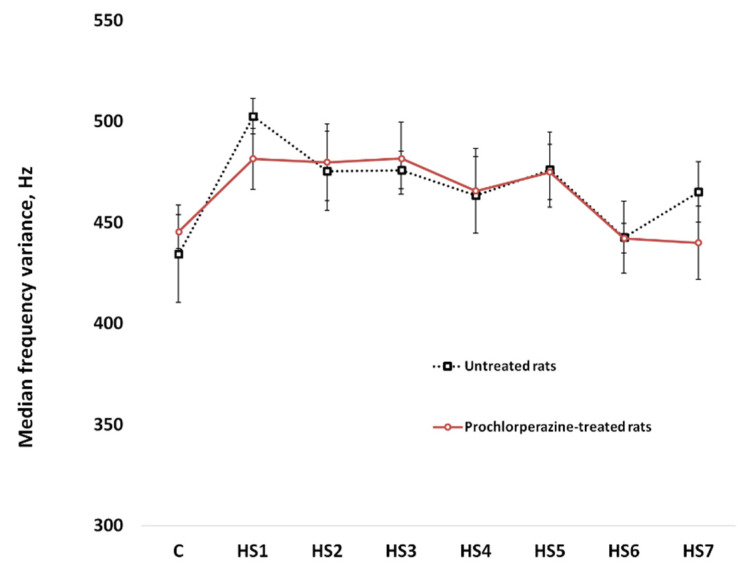
Mean values of median frequency variance during mechanical unloading (hindlimb suspension). C: pre-suspended rats; 1HS–7HS: days of hindlimb suspension. EMG recordings were performed before hindlimb suspension (C) and during 7-day HS in untreated rats and rats treated with prochlorperazine (10 mg/kg daily).

Analysis of the soleus muscle EMG signal also revealed that mean values of the magnitude of the median frequency (Figure 7) and median frequency variance (Figure 8) did not significantly differ between the untreated and prochlorperazine-treated rats during the entire period of mechanical unloading.

## 4. Discussion

About a decade ago, it was found that when the integrity of the spinal cord structure in rats is impaired (spinal cord injury), a significant decrease in KCC2 expression in the motoneurons below the spinal lesion occurs [5,13]. This downregulation of KCC2 leads to an inversion of the chloride current (resulting in a higher neuronal Cl^−^ concentration) and to GABA/glycine receptor-mediated depolarization instead of hyperpolarization [14,15,16]. The decrease in KCC2 expression is considered one of the main reasons for the gradual development of leg muscle spasticity following spinal lesions [5]. Interestingly, Baldwin et al. (2013) pointed out that the neuromuscular changes that occur after spinal cord isolation (a surgical procedure that eliminates neural activation of the motoneurons and associated muscles) are very similar to changes observed following real spaceflight or simulated microgravity (hindlimb unloading) [17]. Under these conditions, an initial drop in the EMG activity of postural muscles (up to 3–4 days of unloading) is replaced by the gradual emergence of electrical activity of rat soleus muscle [1,2,3]. At the same time, under conditions of real and simulated microgravity, both rodents and humans develop hyperreflexia, which indicates an increase in the excitability of motoneurons [18,19].

In the present study, we hypothesized that a decrease in the protein expression of KCC2 in motoneurons could occur under conditions of weight-bearing withdrawal (7-day hindlimb unloading), resulting in an increase in EMG activity in rat postural muscle. Indeed, in the present study, a significant decrease in the total protein content of KCC2 was found in the motoneurons of the lumbar spinal cord after 7-day exposure of rats to unloading conditions. Interestingly, a similar decrease in KCC2 content was detected not only due to spinal cord injury above the lumbar region [5], but also after axotomy as a consequence of peripheral nerve injury [7], i.e., due to impaired motor innervation of skeletal muscles. The data obtained in the present study fully confirm the conclusion of Akhter et al. (2019) that KCC2 expression is determined by the state of neuromuscular control [7]. Moreover, since our data were obtained in an unloading model, characterized by a profound decrease or complete elimination of the neuromuscular activity of rat soleus at the initial stage [1,2,3,20], it can be assumed that KCC2 expression is maintained at a certain level as long as motoneurons receive signals about the normal activity of the innervated muscle. The use of prochlorperazine (KCC2 enhancer) during 7-day mechanical unloading was able to prevent a decrease in KCC2 protein content in spinal motoneurons. A similar effect of prochlorperazine on KCC2 was previously demonstrated in rats after spinal cord injury [9]. Interestingly, in the present study, 7-day HS also led to a decrease in the level of KCC2 Ser940 phosphorylation, which was not, however, prevented by prochlorperazine administration. Our data also show that unloading leads to increased protein expression of NKCC1, a known antagonist of KCC2. Treatment of rats with prochlorperazine was able to prevent the HS-induced increase in NKCC1 content. Thus, we suggest that hindlimb unloading creates all the prerequisites for alterations in chloride (Cl^−^) currents and subsequent inversion of the effect of inhibitory neurotransmitters (glycine and GABA) on spinal motoneurons.

A significant decrease in KCC2 expression in the spinal motoneurons is considered to be the cause of activity and spasticity of the innervated skeletal muscles following spinal cord injury above the lumbar region [5,6,13]. Therefore, we assumed that the emergence of soleus muscle EMG activity under conditions of simulated microgravity (HS) would also be associated with a decrease in KCC2 expression. Indeed, daily prochlorperazine administration during 7-day HS prevented both a decrease in KCC2 content in motoneurons and the emergence of delayed onset EMG activity of rat soleus muscle.

Based on our own data and the available literature, we can propose the following hypothetical chain of events resulting in the emergence and maintenance of tonic neuromuscular activity of the postural soleus muscle under unloading conditions. Inactivation of slow motoneurons due to the elimination of afferent signals from the mechanoreceptors of the feet under mechanical unloading [21] leads to the initial elimination of soleus muscle electrical activity [1,2,3]. Signals about the active state of the muscle (of unknown nature, most likely afferent proprioceptive signals) do not stimulate the corresponding motoneurons in the spinal cord, which leads to decreased KCC2 expression and a subsequent positive shift in the equilibrium potential for Cl^−^. The inhibitory effects of GABA and glycine become more excitatory, resulting in the generation of electrical impulses and the emergence and maintenance of soleus muscle EMG activity during the period of gravitational unloading. It is also noteworthy that the elimination of electrical activity of the soleus muscle under the action of prochlorperazine is accompanied by a decrease in the total protein content of KCC2, but not KCC2 Ser940 phosphorylation, in spinal motoneurons. It appears that the amount of KCC2 molecules, but not their phosphorylation, determines the nature of the chloride current and the subsequent changes in the properties of the spinal motoneurons. Further experiments using other activators/enhancers of KCC2 are necessary to fully confirm the hypothesis regarding the role of KCC2 protein expression in the occurrence and maintenance of the tonic activity of rat soleus muscle under unloading conditions.

To our knowledge, this is the first study showing that delayed onset neuromuscular activity of the rat soleus muscle occurring during 7-day hindlimb unloading is accompanied by a decrease in KCC2 protein expression and phosphorylation in the motoneurons of the lumbar spinal cord. Prochlorperazine administration during 7-day hindlimb unloading prevents a decrease in KCC2 protein expression and significantly reduces the level of electrical activity of the soleus muscle.

## Data Availability

Not applicable.
